# Antibiotic Residues, Antimicrobial Resistance and Intervention Strategies of Foodborne Pathogens

**DOI:** 10.3390/antibiotics13040321

**Published:** 2024-04-02

**Authors:** Yongning Wu, Zhenling Zeng

**Affiliations:** 1 NHC Key Laboratory of Food Safety Risk Assessment, China National Center for Food Safety Risk Assessment, Beijing 100021, China; 2 Research Unit of Food Safety (2019RU014), Chinese Academy of Medical Sciences and Peking Union Medical College, Beijing 100021, China; 3 Guangdong Provincial Key Laboratory of Veterinary Pharmaceutics Development and Safety Evaluation, College of Veterinary Medicine, South China Agricultural University, Guangzhou 510642, China; 4 National Risk Assessment Laboratory for Antimicrobial Resistance of Animal Original Bacteria, South China Agricultural University, Guangzhou 510642, China

## 1. Introduction

The primary determinant of human health is undoubtedly safe food. Nevertheless, unsafe foodstuffs, containing hazardous substances, such as bacteria, chemical, and physical contaminants, at harmful levels can lead to various acute and chronic illnesses, including over 200 diseases ranging from diarrhea to cancers, and even permanent disabilities or death. Alarmingly, an estimated 600 million individuals worldwide—almost one in ten people—suffer from illnesses caused by contaminated food, resulting in a global annual burden of 33 million disability-adjusted life years (DALYS) and 420,000 premature deaths [1].

Given the significance of this issue, it is imperative to monitor potential food safety concerns associated with global changes in food systems, as appropriate, to determine exposure to both new and existing hazards. Food safety science and risk assessment should be used to determine the likelihood of foodborne illnesses. In recognition of this, the World Health Organization (WHO) has updated the Global Strategy for Food Safety, while China has also issued the national roadmap with the One Health Approach [2,3].

The high volume of antibiotics in food-producing animals significantly contributes to the emergence of antibiotic-resistant bacteria (ARB), particularly in scenarios of intense animal husbandry. Notably, in some countries, the total quantity of antibiotics administered to animals surpasses that used in humans by four times. A significant portion of antibiotic use in animals in many countries is aimed at promoting growth and preventing disease, rather than treating sick animals. Resilient bacteria can be transferred from animals to humans through direct contact or via the food chain and the environment. Antimicrobial resistance (AMR) infections in humans can result in prolonged illnesses, increased frequency of hospitalization, and treatment failures that can even lead to death. Tragically, some types of bacteria that cause severe infections in humans have already developed resistance to most or all available therapeutics, leaving us with dwindling treatment options for certain types of infections [4]. 

Food products are increasingly being recognized as important contributors to antibiotic usage, leading to the presence of veterinary drug residues and the transmission of ARB, AMR and their associated ARGs. These unexplained transmission mechanisms pose a significant public health threat to the general population, highlighting the urgent need for effective measures to address this issue. 

Increasing cross-sector connectivity has fueled the global dissemination of ARGs. The concept of ‘One Health’ represents an integrated, unifying framework aimed at harmonizing and optimizing the health of humans, animals and the environment. This approach necessitates collaboration among the public health, veterinary, and public health and environmental sectors. It holds particular significance in ensuring food and water safety, controlling zoonotic diseases, managing pollution from veterinary drug residues, and combatting AMR. In recognition of this urgent need, the WHO initiated a Global Action Plan in 2015 [4], urging for AMR surveillance across these three sectors as well as integrating data to gain insights into the transmission and cross-sectoral connectivity of AMR. Against this global background, China submitted the National Action Plan in 2016–2020 and updated it for 2022–2025, embracing the One Health Approach to tackle AMR surveillance effectively [5] The National Nature Science Foundation of China has kicked off several Major Programs (No 22193060, 32141000, 42021000, 81991535) with the One Health Approach. These efforts are crucial in the global fight against AMR, safeguarding the health and well-being of all. This Special Issue devotes particular attention to the interfaces among humans, animals, plants, food, and the environment, seeking to delve into the intervention strategies used to combat foodborne pathogens. Furthermore, it aims to characterize the composition variations within the healthy human gut microbiome, particularly in relation to antibiotic usage and food consumption. The focus of this Special Issue is to explore food safety intervention strategies involving antibiotics used in animals and plants, while also identifying ARB and ARGs. Through this comprehensive approach, we strive to gain a deeper understanding of these complex interactions and their potential implications for food safety and public health.

## 2. An Overview of Published Articles

Conventional single-level and single-perspective approaches have proven inadequate for the effective prevention and control of AMR and antimicrobial-resistant pathogens. To effectively address this problem, it is necessary to use multidisciplinary and multisectoral cooperation approaches that encompass human, animal, and environmental dimensions. The “One Health” approach offers a comprehensive and systematic framework for tackling AMR and foodborne pathogens from a multidimensional and multifaceted perspective. In this Special Issue of *Antibiotics (Basel)*, a total of 16 papers were published based on the concept and method of “One Health” to control antibiotic residues, AMR and foodborne pathogens. The research fields of these papers cover antibiotic residue detection, AMR generation and spreading, antibiotic pharmacodynamic evaluation and metabolism, discussing various facets of the prevention and control of threats posed by antibiotics and AMR to food safety.

The investigation of antibiotic residue detection includes chloramphenicol, nitrofuran and fluoroquinolone residue on plants and animals. Cao et al. reported that antimicrobials not only affect the production and morphology of mung bean sprouts, but also produce an antimicrobial residue. Additionally, chloramphenicol, enrofloxacin, and furazolidone residue was also found in commercial mung bean sprouts. In a Chinese nation-wide survey, Fei et al. found that the levels of fluoroquinolone residues in chicken were higher than those in pork, with detection frequencies of 3.99% and 1.69%, respectively. Enrofloxacin and its metabolite ciprofloxacin were found to be the most predominant fluoroquinolones. Bor et al. examined pig carcasses for gross pathological lesions and collected pork samples for antibiotic residue testing. Their result showed that the prevalence of antibiotic residues was 41.26% (95% CI, 34.53–48.45%) in Kenya, posing potential public health risks to pork consumers.

The large-scale use of antimicrobials in farms not only increases the potential presence of their residues in food and the environment, but also leads to a greater probability of AMR generation and spread. A major factor in the prevalence of ARGs in the food chain is the presence of ARB in food animals, which poses a potential risk to public health and safety. Han et al. reported the co-occurrence of *optrA* and *cfr*(D) operons in *Streptococcus parasuis* collected from pig farms. The presence of Tn*554*–*optrA* in *Enterococcus* spp., *Staphylococcus* spp., and *Streptococcus* spp. with various genetic and source backgrounds demonstrated that the Tn*554* element plays an important role in the dissemination of *optrA*. This study extended the current knowledge of the genetic background of *optrA* and *cfr*(D) and indicated that Tn*554* and IS*1202* may play an important role in the transmission of *optrA* and *cfr*(D) originating from pig farms. Based on whole-genome sequencing (WGS), a novel plasmid pYhe2001 from swine-origin *Klebsiella pneumoniae* 200 is reported for the first time, suggesting that the plasmids may act as reservoirs for various ARGs and transport multiple resistance genes in *K. pneumoniae* of both animal and human origin.

Colistin is a last-line antibiotic against Gram-negative pathogens. However, the emergence of colistin resistance has substantially reduced the clinical effectiveness of this antimicrobial. In the study of Carhuaricra et al., the occurrence of *mcr-1*-harboring *Escherichia coli* was determined for the first time in chicken farms and pig farms in Lima, Peru. The genomic analysis showed diverse lineages of *E. coli* carrying the *mcr-1* gene mobilized by the IncI2 and IncHI1A:IncHI1B plasmids, including the presence of ISApl1 copies enhancing the dissemination of *mcr-1*. The elevated prevalence of multidrug-resistant (MDR) strains in farms in Lima could serve as a reservoir of ARGs that can be disseminated by farmers or food, impacting public health. A study in Kenya found that poultry meat and pork were contaminated with high levels of bacteria with MDR, potentially spreading foodborne illnesses. This resistance was noted for critically essential antimicrobials (according to the WHO) such as rifampicin (96%), ampicillin (35%), cefotaxime (9%), cefepime (6%), and ciprofloxacin (6%).

The global epidemiological investigation of the AMR of pathogenic bacteria is crucial for clinical therapy and the mitigation of this threat. Colistin resistance in bacteria has become a significant threat to food safety and public health, and its development was mainly attributed to the plasmid-mediated *mcr* genes. Twenty *mcr* variants were identified from 2279 *mcr*-producing *Salmonella* genomes, and the most common ones were *mcr-9.1* (65.2%) and *mcr-1.1* (24.4%). Phylogenetic results indicated that *mcr*-producing *Salmonella* fell into nine lineages (Lineages I-IX), and *Salmonella* Typhimurium, 1,4,[5],12:i:- and 4,[5],12:i:- isolates from different countries were mixed into Lineages I, II and III, suggesting that international spread occurred in *Salmonella*-bearing *mcr* genes. Liu et al. examined the synergy between colistin and capric acid against twenty-one Gram-negative bacterial isolates. Checkerboard and time–kill assays showed that capric acid can enhance the bacterial killing of colistin-resistant Gram-negative bacteria when combined with colistin.

The widespread escalation of bacterial resistance threatens the safety of the food chain. While investigating the resistance characteristics of *E. coli* strains isolated from disinfected tableware against both disinfectants and antibiotics, a recently described mobile colistin resistance gene *mcr-10* present on the novel IncFIB-type plasmid was found to be able to successfully transform the resistance. This work warned that continuous monitoring of ARGs in the catering industry is essential to understand and respond to the transmission of ARGs from the environment and food to humans and clinics. Shared bikes act as a potential vector for ARB and ARGs. Two ST167 *E. coli* isolated from shared bikes show high similarities in their core genomes and plasmid profiles with strains from hospital inpatients and farm animals. This study indicated that vectors such as shared bikes may contribute to the dissemination of these ARB in the environment. There is a need to take measures to assess the risk of ARB in the environment and cut off transmission.

The emergence of the mobile tigecycline-resistance gene, *tet(X4)*, poses a significant threat to public health. The study of Zhai investigates the prevalence and genetic characteristics of the *tet(X4)*-positive *E. coli* in clinical human stool samples. The clonal spread of *tet(X4)*-positive isolates indicated the risk of intra-hospital transmission of the *tet(X4)* gene. Furthermore, these strains and plasmids of clinical patient origin showed a strong genetic resemblance to some animal-origin strains, implying a potential risk of transmission between animals and humans.

The growing concern over the emergence of AMR in animal production as a result of extensive and inappropriate antibiotic use has prompted many swine farmers in Canada to raise their animals without antibiotics (RWA). Alvarado et al. investigates the impact of implementing an RWA approach in sow barns on actual on-farm antibiotic use, the emergence of AMR, and the abundance of pathogens. Metagenomic analyses demonstrated an increased abundance of pathogenic *Actinobacteria*, *Firmicutes*, and *Proteobacteria* in the nasopharynx microbiome of RWA sows relative to non-RWA sows. WGS analyses revealed that the nasal microbiome of sows raised under RWA production exhibited a significant increase in the frequency of resistance genes coding for β-lactams, MDR, and tetracycline.

Antibiotic usage and yogurt consumption are the major interventions for gut microbiota. Yan et al. found that antibiotic usage and yogurt consumption demonstrated significant changes in specific bacterial groups (*Streptococcaceae*, *Enterococcaceae* and so on) in healthy human gut microbiomes, sharing more identical changes in the healthy human gut microbiome than disparities, especially ARG-related bacteria groups that could induce an intensification of ARG transfer processes from commensal bacteria to pathogens in the human gut.

Phorate is a systemic, broad-spectrum organophosphorus insecticide. Cao et al. assessed the blood glucose concentrations of high-fat-diet-fed mice exposed to phorate and the distribution characteristics of the resistance genes in the intestinal microbiota of these mice. The result revealed that phorate can affect the abundance of the intestinal microbiota and therefore alter the expression of drug-resistance genes. This study indicates that changes in the abundance of the intestinal microbiota are closely related to the presence of ARB in the intestinal tract and the metabolic health of the host.

In the study of Mao et al., the metabolism behavior of mequindox (MEQ) in sea cucumber in vivo was investigated using LC-HRMS. This work first reported 3-methyl-2-quinoxalinecarboxylic acid (MQCA) as a metabolite of MEQ, and carboxylation is a major metabolic pathway of MEQ in sea cucumber. This work revealed that the metabolism of MEQ in marine animals is different from that in land animals. The metabolism results in this work could facilitate the accurate risk assessment of MEQ in sea cucumber and related marine foods.

## 3. Conclusions

AMR poses a significant threat to human health. This compilation of articles dedicated to the field of retailing encompasses a comprehensive overview of a diverse range of research, elucidative of the richness of the research field. The articles showcase a range of methodologies that were adopted for the studies of occurrence and genomic characterization of AMR, ranging from qualitative approaches based on in situ observations and interviews to quantitative studies using omics and artificial intelligence (AI) tools to trace the evolution of bacteria harboring certain emerging ARGs or/and mobile genes. Case studies focus on specific ARGs and plasmids, such as the colistin resistance gene *mcr-X*, *bla*_NDM-5_ and *bla*_CTX-M-199_, *tet(X4)-IncX1*, *cfr*(D) and *optrA*, providing insights into their dissemination and health impact. Notably, many bacteria carried by animals (such as *Salmonella*, *Campylobacter* and *E. coli*, and *K. pneumoniae*) can also cause diseases in humans. These bacteria, often harboring ARGs, can contaminate our food supply throughout the entire production chain, from farm to fork, during slaughtering and processing. Vegetables, including mung bean sprouts, as described in this Special Issue, are susceptible to contamination by harmful bacteria, either at the farm or subsequently through cross-contamination. This knowledge is derived from our ability to trace the origin of ARB isolated from ill individuals back to agricultural sources using DNA fingerprinting techniques. This underscores the importance of vigilant monitoring and sanitary practices throughout the entire food production chain to ensure food safety and protect public health.

Annually, over 400,000 people die from foodborne diseases, with children under five years old accounting for over a third of these tragic deaths. According to estimates by the WHO, microbes, including bacteria, cause the vast majority of foodborne illnesses [1]. Alarmingly, if these bacteria develop resistance to antibiotics, effective treatments will become limited, leading to an increase in deaths from foodborne diseases. Therefore, optimizing the use of antibiotics in both human medicine and animal husbandry is crucial to mitigate the emergence and spread of ARB and ARGs, thereby safeguarding public health. To address the critical public health threat, the WHO has developed several guidelines aimed at preserving the effectiveness of antibiotics vital for human health. Our recommendations align with the WHO’s list of critically important antimicrobials for humans, especially critically important antibiotics, to treat multidrug-resistant infections in humans. When considering antibiotics used in food-producing animals, it is essential to prioritize those with the least significance for human health. This means starting with antimicrobial classes that are not used in humans, and then proceeding with those listed on the WHO’s list of critically important antimicrobials for human medicine, followed by those classified as highly important. Antibiotics categorized by the WHO as critically important for human medicine should be used in animals only when the most recent culture and sensitivity results of bacteria known to have caused the disease indicate that this critically important antimicrobial is the sole viable option. Competent authorities may require a cross-disciplinary “One Health” approach when evaluating new hazards emerging at the human–animal–environmental interface [6]. This approach can be instrumental in minimizing the use of antibiotics in food animal production by enhancing husbandry and management practices for disease prevention and control, as well as strengthening AMR surveillance within the food chain. By adopting these strategies, we can work towards mitigating the threat of AMR and safeguarding public health.

The WHO has established a “One Health Initiative” to integrate efforts in humans, animals, and environmental health across its organization. Given the interconnectedness of human and animal health, it is crucial to incorporate information gathered from pathogens in animals and the food chain into AMR surveillance programs, which falls under the umbrella of the One Health framework. The WHO is collaborating with the Food and Agriculture Organization of the United Nations (FAO), the United Nations Environment Program (UNEP), and the World Organization for Animal Health (WOAH) as part of a One Health quadripartite [7]. This quadripartite promotes multi-sectoral approaches aimed at reducing health threats at the intersection of humans, animals, and the ecosystem. The quadripartite One Health Joint Plan of Action (OH-JPA) outlines the necessary transformations to prevent and mitigate the impact of current and future health challenges at the global, regional, and country levels. Notably, AMR and food safety are included in this plan. However, according to the latest global database from the Tracking AMR Country Self-Assessment Survey (TrACSS, 2023) [8], which has been executed in 177 countries, only 16 countries have formalized multi-sectoral coordination mechanisms with functional working groups, and only 25 countries possess adequate technical capacity, resources, and established systems to gather data across the “One Health” sectors. This highlights the urgent need for further development and coordination to fully realize the potential of the “One Health” approach in addressing AMR and other critical health challenges.

In high-income countries, trends in AMR in animals and food are monitored via systematic surveillance by organizations such as the European Food Safety Authority (EFSA) in Europe [9], the National Antimicrobial Resistance Monitoring System for Enteric Bacteria (NARMS) in the United States [10], or the Canadian Integrated Program for Antimicrobial Resistance Surveillance (CIPARS) in Canada [11]. The One Health approach can also be found in a UK report [12]. However, in low- and middle-income countries (LMICs), where demand for meat (and antimicrobials) is rising, rapid systematic surveillance systems remain largely absent. This Special Issue seeks to promote this approach further.
